# The nature of photoinduced phase transition and metastable states in vanadium dioxide

**DOI:** 10.1038/srep38514

**Published:** 2016-12-16

**Authors:** Zhensheng Tao, Faran Zhou, Tzong-Ru T. Han, David Torres, Tongyu Wang, Nelson Sepulveda, Kiseok Chang, Margaret Young, Richard R. Lunt, Chong-Yu Ruan

**Affiliations:** 1 Department of Physics and Astronomy, Michigan State University, East Lansing, Michigan 48824 USA; 2 Department of Electrical and Computer Engineering, Michigan State University, East Lansing, Michigan 48824 USA; 3 Department of Chemical Engineering and Materials Science, Michigan State University, East Lansing, Michigan 48824 USA

## Abstract

Photoinduced threshold switching processes that lead to bistability and the formation of metastable phases in photoinduced phase transition of VO_2_ are elucidated through ultrafast electron diffraction and diffusive scattering techniques with varying excitation wavelengths. We uncover two distinct regimes of the dynamical phase change: a nearly instantaneous crossover into an intermediate state and its decay led by lattice instabilities over 10 ps timescales. The structure of this intermediate state is identified to be monoclinic, but more akin to M_2_ rather than M_1_ based on structure refinements. The extinction of all major monoclinic features within just a few picoseconds at the above-threshold-level (~20%) photoexcitations and the distinct dynamics in diffusive scattering that represents medium-range atomic fluctuations at two photon wavelengths strongly suggest a density-driven and nonthermal pathway for the initial process of the photoinduced phase transition. These results highlight the critical roles of electron correlations and lattice instabilities in driving and controlling phase transformations far from equilibrium.

Phase transitions are often discussed within the context of thermal equilibrium where distinct phase change phenomena are described in the complex phase diagram in which temperature, chemical doping, or pressure are steady-state tuning parameters. Much less is understood in terms of excited states and dynamical phase transitions induced after sudden excitations using intense laser pulses^1^,^2^. In this strongly nonequilibrium condition, the thermodynamic ground state may become less stable than its counterparts and metastable new phases may emerge^3^,^4^. Characterizing the nonequilibrium transformations between two thermodynamic phases directly or through a metastable phase indirectly may provide valuable insights regarding the nature of these states, the interactions, and the active coordinates involved in these phase transitions^5^.

As a prime example of phase transitions, the metal-to-insulator transition in VO_2_ has attracted wide-spread attention and is one of the best studied at the ground states near equilibrium. Pristine VO_2_ crystals undergo a first-order transition from a high-temperature metallic phase with a rutile (R) structure to a low-temperature insulating phase with a monoclinic (M_1_) structure at the transition temperature *T*_*c*_ = 340 K^6^,^7^,^8^. The introduction of electron^9^ and hole^10^ doping changes the transition temperature, as well as leads to the appearance of new phases such as M_2_ and T, resulting in a complex phase diagram as illustrated in Fig. 1(a). To decipher the apparent synergy between the electronic and lattice degrees of freedom culminating to a large-scale electronic switching^11^,^12^, scenarios involving both Mott-Hubbard^10^,^13^ and Peierls^14^,^15^ physics were proposed, and their verifications have been the subjects of intense experimental investigation, particularly using ultrafast pump-probe approaches^15^,^16^,^17^,^18^,^19^,^20^,^21^,^22^ to transiently isolate the active components.

Among the established work, ultrafast insulator-metal transition (IMT) has been the early focus using ultrafast optical pump-probe techniques. A view based on the temporal hierarchy suggested a nonequilibrium nature of transformations, judged from the ~100 fs timescale optical responses^22^,^23^,^24^ and x-ray absorption changes^25^, which clearly is decoupled from the structural transitions expected to proceed on ps electron-phonon coupling or longer timescale. This ultrashort temporal response in optical properties may reflect the differences in heat capacity in electronic and lattice degrees of freedom, thus yielding different response times in suppressing the respective order parameters (optical gap and diffraction intensity) through coupling between transiently isolated sub-systems^26^,^27^. However, through pump-fluence dependent studies, the evidences of decoupling on the long-range scale between electronic and structural phases were demonstrated in the observation of a threshold for the onset of IMT to be ≤ 1 mJ/cm^2^ 20,24, in distinct contrast to the onset fluence of 4–8 mJ/cm^2^, identified for the monoclinic–to-rutile structural changes using electron^16^,^27^,^28^ and x-ray^17^ diffraction or from persistent THz conductivity^20^,^21^. From the viewpoint of displacive excitation of coherent phonons^29^, the interplay between the electronic and lattice degrees of freedom were examined. Notably, structural bottleneck in IMT is proposed based on a minimum transition time of ~75 fs, which corresponds to one-half period of the *A*_*g*_ phonon modes that stretch and twist the V-V dimers as the active modes driving the electronic changes^15^. More detailed classification of the modes^18^,^20^,^30^ and their role as active modes pertaining to insulating or metallic phase^30^, as well as emerging anisotropy near critical temperature^26^, were reported, suggesting the involvements of different phonon modes during the phase transitions. In particular, two distinct symmetry changes involving the initial steps of monoclinic-to-rutile transformation were identified through ultrafast electron diffraction^16^,^28^. Meanwhile, in the strongly driven regime, a sub-60 fs band-gap collapse breaking the structural bottleneck timescale was demonstrated in ultrafast photoemission studies, implying the dominant energy scale at the Fermi energy being the electron correlations^22^,^31^. Given the temporal hierarchy and the distinct thresholds between the electronic and structural transitions, a new monoclinic metallic phase that bridges the two transition has been suggested^18^,^27^ and their presence as a long-lived metastable state induced under photoexcitations^28^,^32^,^33^, or induced under strong stress^34^,^35^ and/or carrier doping^27^,^35^ were increasingly reported. Identifying the emergence of such a new state and clarifying its role in mediating the electronic and structural transformations are of major interests to address key open questions regarding the apparent complexities involved in the phase transition of VO_2_.

In this work, we present controlled studies to examine the transient emergence of new metastable phases and their nonequilibrium dynamics during photoinduced phase transitions of VO_2_ using femtosecond electron diffraction^16^,^36^,^37^ and diffusive scattering techniques, which allow us to directly monitor the momentum-dependent lattice dynamics. With different wavelengths of optical pumping, we demonstrate that the photoinduced phase transitions in VO_2_ is non-thermal, evidenced by the fact that the optical threshold of the phase transition is best described in terms of photodoping rather than the energy density. By investigating the transient diffraction patterns, we discover a unique interaction-driven monoclinic emergent state that, interestingly, has a similar crystal symmetry as the thermodynamic M_2_ phase. Our results show that the photoinduced phase switching between the insulating M_1_ and the metallic R phases can be significantly more efficient by accessing the intermediate phase and the new correlation-driven pathways.

## Results and Discussion

**Figure 1:**
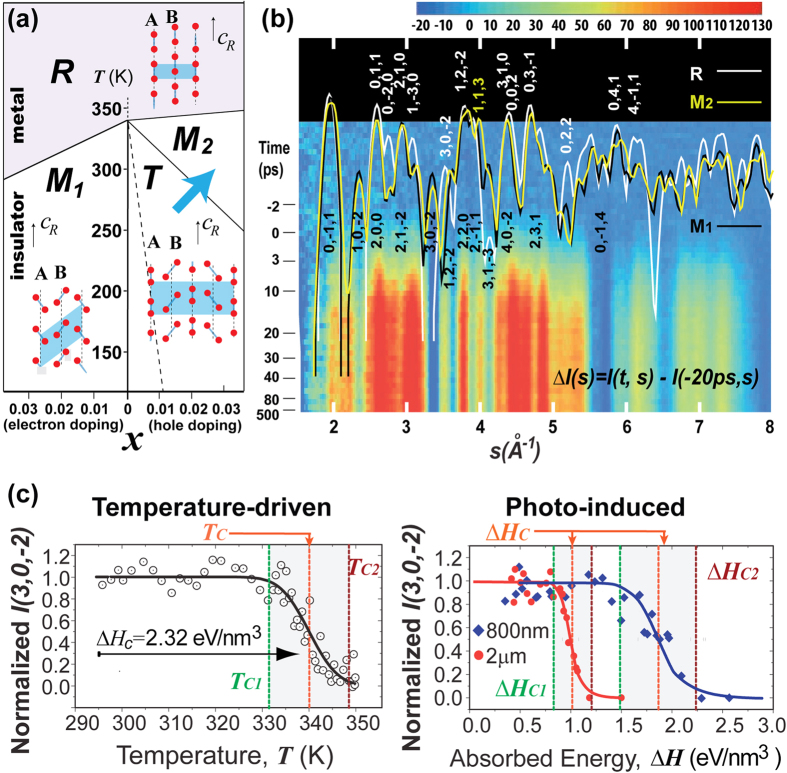
Phase transitions of VO_2_. (**a**) Layout of the phase diagram of VO_2_, along with the schematic diagram of structural displacements in each phase. (**b**) The time-dependent diffraction curves from VO_2_ films driven by 800 nm pulses, presented in difference map (reference −20 ps). The simulated diffraction curves, in log scale along with the corresponding Miller indices, based on known crystalline phases are overlaid atop the difference map for comparison. (**c**) The M_1_-to-R transition curves under the steady-state heating (black) and under excitations of near-IR (800 nm, blue) pulses, mid-IR (2000 nm, red) pulses. The solid curves are error function fittings to derive the critical energy density Δ*H*_*C*_ and transition window specified by Δ*H*_*C*_ ± 2σ. The initial sample temperature is 294 K.

In our experiments, we employed ultrafine films of VO_2_ nanocrystalline grains (size 31 ± 7 nm) prepared on a 9 nm amorphous Si membrane using vapor deposition method (see Methods). The grain size and nanofilm settings were chosen to leverage their flexibility on the nanometer scale to avoid excessive stress^27^,^38^ that may otherwise develop during disruptive structural transitions. These samples display high crystallinity from clear crystalline powder diffraction (see Supplementary Fig. S1). Using the ultrafast electron diffraction setup^5^ (see Methods), the patterns of time-dependent diffraction are recorded in the difference map in Fig. 1(b), where the patterns from different known VO_2_ crystalline phases^39^,^40^,^41^ are also shown. Sufficiently low repetition rates (≤500 Hz) were employed to avoid irreversible changes [see Supplementary Information (SI) for details]. The major sequence of events associated with symmetry-recovery phase transition from M_1_ to R can be identified based on the evolution of relative Bragg intensities. Such evolution is sensitive to changes in the two critical lattice distortions (pairing and twisting) along the V-V sub-lattice chains (A and B), as depicted in Fig. 1(a). For example, the ultrafast transformation of the distorted M_1_ is measured by diminishing pairing-specific reflections [e.g. (3, 0, −2), (1, 0, −2), (3, 1, −3)] in the early transition, whereas at the later times formation of the undistorted R is confirmed from specific enhancements and movements of the high-symmetry peaks.

**Figure 2:**
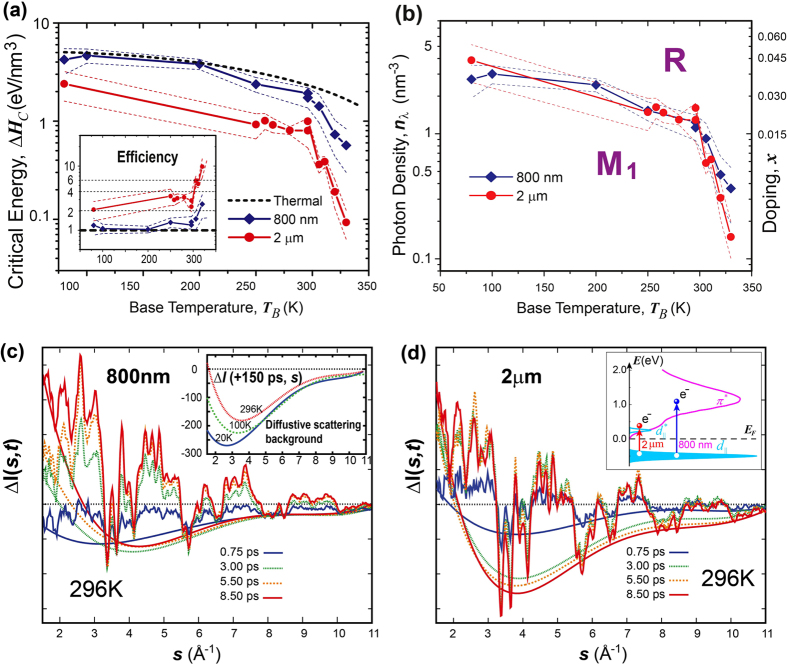
Phase transitions of VO_2_ under optical pumping. (**a**) The transition critical energy density, Δ*H*_*C*_ (solid) and Δ*H*_*C*_ ± σ (dash), as a function of crystal temperature T_B_ under two different optical excitations. The thermal critical threshold (dash, black) is also plotted for comparison. The inset in (**a**) shows the efficiency based on normalizing the Δ*H*_*C*_ associated with the photoinduced transitions obtained for each *T*_*B*_ with the corresponding Δ*H*_*C*_ from the thermal transition. (**b**) The optical doping – temperature phase diagram, converted from (**a**), where the photon density *n* = Δ*H/E*_*λ*_, the doping *x* = *n·V*_*R*_, and the VO_2_ formula volume *V*_*R*_ = 2.96 × 10^−23^ cm^3^. (**c**,**d**) The diffraction difference curves Δ*I*(*s, t*) obtained by subtracting the reference ground state curve (−20 ps) from the time-dependent diffraction curve *I*(*s, t*). To highlight the trend of the diffusive scattering evolution, curves are obtained by fitting the baseline line using a 4^th^ order polynomial function via maximizing the non-negativeness of Δ*I*(*s, t*) (see text). The inset in (**c**) shows the temperature-dependent diffusive scattering curves obtained at 150 ps. The inset in (**d**) illustrates the interband transitions induced by 800 nm and 2 μm pump lasers from the *d*_||_ band to the π^*^ band of VO_2_. The density of states qualitatively reproduce the results in ref. 11.

Our controlled studies were based on femtosecond (45 fs) infrared (IR) pumping using two different photon wavelengths, 800 nm and 2 μm (photon energy *E*_*λ*_ = 1.55 and 0.62 eV respectively), through which different energy densities were absorbed by the VO_2_ crystals to produce a similar number of electron-hole pairs. The transition curves presented in Fig. 1(c) were obtained by incrementally increasing the laser fluence while observing the intensity of order-parameter reflection (3, 0, −2) at +150 ps delay. Indeed, using near-IR (800 nm) pump pulses we observed clear threshold switching behavior with the transition at a critical fluence *F*_*C*_ ~ 7 mJ/cm^2^ (determined by fitting with the error function) at room temperature. This is within the range of previously identified thresholds (4–8 mJ/cm^2^) also under 800 nm photoexcitation^16^,^19^,^20^,^22^,^27^,^28^,^30^,^31^. We note that in order to precisely determine the pump-laser fluence, we measure the pump-laser spot size *in situ* by the knife-edge and laser-electron cross-correlation measurements (see Methods and SI). The error of fluence measurements introduced by laser-spot size measurements is no more than 25%. To compare with thermal transition, the absorbed optical energy density Δ*H* is calculated from the applied fluence *F* by Δ*H* = *Fγ*/*d*, where *d* is the film thickness. The absorption coefficient *γ* is carefully determined through measuring the wavelength-dependent transmittance and reflectance using the transfer matrix method on the as-grown VO_2_ films (see SI for details). Based on *F*_*C*_, the critical energy dose Δ*H*_*C*_ for 800 nm pump is ~2 eV/nm^3^ which is close to the thermal requirement (2.32 eV/nm^3^) for transition between M_1_ and R^42^, calculated based on integrating heat capacity and latent heat (see SI for details). This similarity between Δ*H*_*C*_ estimated from photoinduced and thermal transitions has been the reason behind considering the near-IR-driven phase transition as quasi-thermal in the established work. However, we show that the mid-IR (2 μm) pumping leads to a much smaller critical dose (Δ*H*_*C*_ ~1 eV/nm^3^). This unexpected result is demonstrated over a range of temperatures [Fig. 2(a)], where we consistently observe a subthermal critical dose under mid-IR whereas the transition under near-IR occurs close to the thermal threshold. However, this disparity may be reconciled by converting the critical energy density to the absorbed photon density *n*_*C*_ = Δ*Hc/E*_*λ*_ depicted in Fig. 2(b). The critical lines separating M_1_ and R as defined by *n*_*C*_ determined using the two optical excitations then agree quite well. This result thus indicate that the fundamental process of photoinduced phase transitions is very different from a thermally induced transition, and strongly suggests a density-driven mechanism at play in the photoinduced phase transition between M_1_ and R phases^1^.

We note that our results are distinctly different from the conclusions drawn in ref. 24, which we believe can be attributed to the difference in experimental approaches. In ref. 24, the optical response shortly after pump excitation (+200 fs) was monitored leading to the reported thresholds of the absorbed energy density several times smaller than our results and other work^16^,^18^,^19^,^20^,^21^,^28^,^30^. Indeed, as shown in previous THz spectroscopy studies^18^,^19^,^20^,^21^, even though the optical conductivity is increased by laser-induced photodoping in ~100 fs, such increment decays within 1 ps so that it does not persist to induce a full phase transition in VO_2_. In our experiments, however, we monitor the VO_2_ structure at a time well after pump laser excitation (+150 ps) in order to observe the threshold of the full photoinduced phase transitions.

The discovery that the mid-IR-driven structural transformation is significantly subthermal has direct ramifications in understanding the unconventional features of photoinduced phase transitions in VO_2_. In particular, the fact that Δ*H*_*C*_ required for driving transition from M_1_ to R by pumping VO_2_ with the mid-IR photons is even below the thermal latent heat (1.47 eV/nm^3^)^42^,^43^ [Fig. 2(a)] suggests a very different pathway that does not involve significant entropy and that may be facilitated by strongly interacting electrons similar to the pathway of chemical doping as elucidated by the phase diagram [Fig. 1(a)]. In such a scenario, the photoexcitation explores excited energy landscape with new emergent properties as defined by a reconstructed energy landscape induced by photodoping, which are not the same as those pertaining to the temperature-driven phases^3^,^4^,^5^. Nonetheless, thus far, the understanding of the emergent state properties focuses on the effect of photodoping, whereas the frequently used near-IR pulses (*E*_*λ*_ = 1.55 eV) not only excite charge carriers that promptly induce doping of correlated electron orbitals, but also impart their excessive photon energy (Δ = *E*_*λ*_ − *E*_*g*_, *E*_*g*_ = 0.6 eV is the optical gap^44^,^45^) which can translate to the lattice as stochastic heating at a timescale of 10 ps and longer. The application of mid-IR pulses (0.62 eV) thus represent an important test scenario where at the same level of charge carrier excitation, their initial excessive photon energy is reduced by nearly an order of magnitude compared to near-IR excitations. To carefully evaluate the impact of both doping and thermal effects, we resort to directly observing the details of the nonequilibrium dynamics under two different photon wavelengths. To follow the coherent structural evolution driven by the sudden electronic impact, we examine the time-dependent coherent Bragg diffraction. Meanwhile, to gauge the role of lattice instabilities and thermal fluctuations we monitor the diffusive scattering away from the Bragg peaks.

Given the short wavelength of the electron beam, the large Ewald sphere associated with ultrafast electron diffraction experiments provides an easy access to short and long-range scale dynamics manifested in both Bragg diffraction and diffusive scattering channels in the reciprocal space that are simultaneously recorded with an area detector. First, we examine the lattice structural evolution during VO_2_ phase transition, where the critical lattice distortions (pairing and twisting) along the chains A and B [Fig. 1(a)] are at the level of 7% of the interatomic V-V distance (~2.8 Å) or less. These subtle changes lead to only a few percent adjustments of key Bragg peak intensities and are best described in the diffraction difference curve Δ*I*(*s, t*) at different pump-probe time delays (*t*) as depicted in Fig. 2(c) and (d). Here, *ΔI*(*s, t*) = *I*(*s, t*) − *I*(*s, t* < 0), where *I*(*s, t*) is the 1D scattering wave-vector (*s*)-dependent pattern reduced by radially averaging the 2D powder diffraction patterns as shown in the Supplementary Fig. S2(b). We note Fig. 2(c) and (d) are obtained using two different pump lasers (800 nm and 2 μm) set at the upper density threshold [*n*_*c2*_ = Δ*Hc*_*2*_*/E*_*λ*_, see Fig. 1(c)], but differing in energy density delivered to VO_2_ nanocrystals. It is intriguing to see that despite of very different excess energy imparted in VO_2_ crystals, the ultrafast timescale relative changes in the Bragg diffraction show nearly synchronous evolution between the near-IR and mid-IR excitations. These ultrafast cooperative evolutions feature the suppression of pairing-specific peaks and the transfer of scattering weight to higher symmetric peaks. These processes describe a transition from M_1_ to a new state with lower lattice distortions being induced by carrier excitations rather than heating.

Next, we turn our attention to the evolution of diffusive scattering (DS) in the region between the Bragg peaks. In the steady-state investigation, DS has traditionally been the source of information for probing the lattice fluctuations and inhomogeneous strain^46^, and in the case of 2D diffraction from nanocrystals, such signals are isotropically integrated, forming a smooth background underneath the Bragg peaks. In ultrafast diffraction, the time-independent common background of *I*(*s, t* < 0), predominantly consisted of incoherent features such as the atomic background and elastic scattering from atomic impurities, are effectively removed in Δ*I*(*s, t*). Therefore, the observed background changes represent the photoinduced features, including inelastic phonon scattering and inhomogeneous strain that evolves as a function of time after pump-laser excitation. Two distinctive dynamics differentiated by their momentum transfer (*s*) and timescale are observed. First, in proximity to the Bragg peaks, the diffusive background exhibits sub-ps suppression. This feature correlates well with an ultrafast shift in the bonding state featured also in the recovery of local symmetry witnessed earlier in the Bragg diffraction on a similar timescale. Here, the suppression of DS represents a reduction of non-uniform strain present in nanocrystals, as part of global symmetry recovery dynamics. Meanwhile, we observe an increase of DS at the small *s* (in regions below the coherent Bragg diffraction) that continues over ps timescales. This enhanced DS at small *s* signifies the emergence of unique longer wavelength correlations, which is highly dependent on the excitation wavelength. We can correlate the contrasting long-wavelength DS in Fig. 2(c) and (d), drawn on the same relative scale, with the different applied photon excess energies. Much larger increase is seen on ~10 ps timescale under 800 nm excitation than under 2 μm excitation. The correlation between such DS and crystal temperature can also be established from the temperature-dependent studies [see inset of Fig. 2(c)], where such an enhancement is reduced at lower temperature under the same optical dose (Δ*n*_*c2*_) over a longer time (+150 ps). Note, to guild the eyes, the overall trend of DS can be followed through the curves underneath the Δ*I*(*s, t*), and those presented for the temperature-dependent studies, generated through a 4^th^ order polynomial fit of Δ*I*(*s, t*) via optimizing the non-negativeness – a practice also used in isolating the incoherent scattering background contribution in powder diffraction curves^47^,^48^. The fact that a significantly lower level of lattice instabilities was found when the mid-IR pump was applied compared to the near-IR one suggests a much lower energy dose being transferred to the lattice. These clear wavelength-dependent results support that the initial photoinduced phase transition is indeed nonthermal.

**Figure 3:**
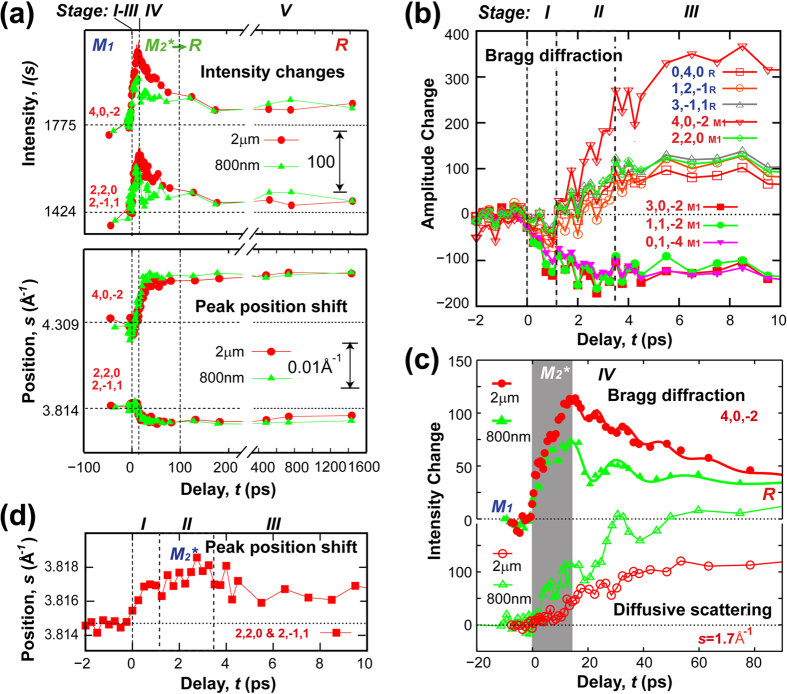
Ultrafast evolutions of Bragg diffraction and diffusive scattering. (**a**) The intensity and position evolutions of the selected Bragg peaks during structural phase transition from M_1_ to R. The laser doses for two different excitations (800 nm and 2 μm) are set to *n*_*c2*_ ~ 1.85 nm^−3^, which is above the transition critical dose (*n*_*c*_ = 1.25 nm^−3^) at crystal temperature *T*_*B*_ = 294 K. (**b**) The scale-up view of the transitions for the first 10 ps, during the formation of metastable phase M_2_*, under 800 nm excitation, including both low-symmetry pairing specific and higher-symmetry reflections. (**c**) The scale-up view of the transitions in the intermediate timescale where the decay of metastable phase occurs. The evolution of diffusive scattering amplitude monitored at *s* = 1.7 Å^−1^ are also depicted for comparison. (**d**) Scale-up view of the center-of-mass position evolution of the (2, 2, 0) & (2, −1, 1) diffraction groups.

To further identify the density-driven state featured by the coherent structural evolution, we examine the transient evolution of the intensity and position of phase-sensitive Bragg peaks over *s* = 3.5 to 4.5 Å^−1^ (Fig. 3). These changes are monitored in reference to the initial (M_1_) and final (R) state parameters both in the longer [Fig. 3(a)] and in the ultrafast [Fig. 3(b)] timescales. Judging from the Bragg peak intensity changes [(4, 0, −2) and (2, 2, 0)/(2, −1, 1) in Fig. 3(a)], we find that the transition into a metastable phase, which is neither M_1_ nor R, is established following initial cooperative structural evolutions (stages I & II, to be discussed later), as further detailed in Fig. 3(b) along with several other reflections. The metastable phase starts its relaxation into R at a well-defined onset time ~15 ps as characterized by a sharp drop of (4, 0, −2) and (2, 2, 0)/(2, −1, 1) intensity (stage IV) as depicted in Fig. 3(c). But the changes are different over two different excitations, in particular with regard to generation of acoustic oscillations that are seen in the modulation of the Bragg intensities. Scenarios to a stable excited electronic manifold due to the soft-band effect in the narrow-gap Mott insulator VO_2_ have recently been proposed theoretically^49^. In our experiments, the above-the-gap photoexcitations inject electrons into the spatially extended π* (V*3d*-O*2p*), and leave holes in the localized spin-singlet *d*_*||*_ (V*3d*-V*3d*) orbitals (see inset of Fig. 2(d))^8^,^11^, effectively prompting hole doping. Given the spatially decoupled orbital symmetry between these states that weakens excitonic coupling^50^, the long-lived excited charge carriers can drive ps timescale structural evolution into a new state which is a local minimum in the excited energy landscape. Furthermore, given the strongly interacting nature^44^,^51^ the evolution may proceed beyond the rigid-band picture^11^,^12^, and establish a new metastable state. This correlation-driven dynamics for reconstructing the electronic energy landscape can be reconciled with the density-driven characteristics as witnessed here, and more specifically the relevant energy scale for triggering the phase transition is set by the hole doping concentration defining the well-established threshold behavior^5^. Meanwhile, with the elevated transient photo-carrier concentration (up to ~10^21^ cm^−3^) the emergent state could exhibit metallic transmissivity^28^ and THz conductivity^18^,^20^, despite that it is not in a true metallic ground state.

According to our results shown in Fig. 3, there appears to be no direct pathway connecting M_1_ to R over the excited landscape in the density-driven regime. The cooperative structural evolutions into the metastable phase presented here were obtained at the same doping level *n*_*c2*_, which is ~20% above the mean threshold of the transition. Meanwhile, an excitation-wavelength-dependent contrasting build-up of the long-wavelength diffusive scattering [Fig. 3(c)] on the timescale of the decay reflects the different applied photon energies translating into lattice instabilities. The relaxation of the emergent state, which, according to its correlation to the diffusive scattering changes, can be associated with instability-driven decay of charge carriers. The process is disruptive enough to initiate strain waves manifested in the breathing mode of VO_2_ nanograins, as observed in the oscillations of the diffraction intensity in Fig. 3(c). The oscillations are more pronounced when driven by the near-IR photons, which also leads to a shorter decay time (6 ps), as compared to the mid-IR pump (40 ps). The timescales are fitted with a function of exponential decay modulated by chirped harmonic oscillations. The oscillation period shifts from ~13 ps at the onset to ~25 ps near the end of each decay, suggesting a varying sound speed from 4.5 km/s to 2.5 km/s (based on breathing mode over ~30 nm grains). It is worth to mention that the lattice softening leading to a similar change of the sound velocity during monoclinic-to-rutile phase transition was also observed previously^52^. Given that the supporting ultrathin Si film for growing the VO_2_ is amorphous, it is expected that the sample-substrate strain mismatch as shown in previous work^34^ does not contribute to our results. Therefore, we expect the evolution of the strain is intrinsic to the VO_2_ nanocrystal induced during phase transitions. It is also worth noting that the onset of the transition correlates well with a build-up of long-wavelength fluctuations near unitcell distance or longer (~2π/*s*_*C*_, *s*_*C*_ ≥ 1.7 Å^−1^) revealed in the diffusive scattering [Fig. 3(c)], suggesting median range lattice fluctuations to be effective in facilitating carrier relaxations.

**Figure 4:**
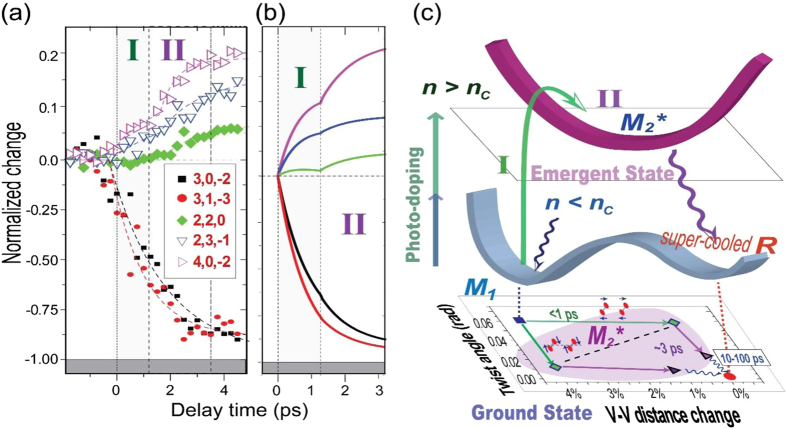
Ultrafast transformation pathways of VO_2_. (**a**) The integrated intensity changes based on the data presented in Fig. 3 (with diffusive background subtraction) under 800 nm excitation. The dashed lines are drawn as a guide to the eyes. (**b**) Simulated intensity changes based on a two-step transition model along the pathways to reduce the lattice distortions along two critical modes [coordinates depicted in (**c**)]: (I) from M_1_ to M_2_^*^ (transition state), and (II) from M_2_^*^ towards R. (**c**) Conceptual depiction of interaction energy landscape in the multistep phase evolutions along with a plot depicting the corresponding time steps and changes along de-pairing and detwisting coordinates estimated from the experimental data.

The energy-dependent nonequilibrium dynamics reported here elucidate the exceptional nature of the density-driven photoinduced phase transitions in VO_2_, which may be similar to the photoinduced insulator-metal transition involving charge-density wave transformation in TaS_2_^5^. In particular, the density-driven emergent state offers a new pathway to reach a stable R phase by circumventing the energy and time consuming entropic barrier. Regarding the identity of the emergent state, we conduct structure analysis based on the integrated intensity evolution leading to such a phase, as depicted in Fig. 4(a) [compared to Fig. 3(b), the intensity is integrated over the Bragg peak envelop after removing the scattering background]. The transition shown in the evolution of (2, 2, 0), (2, 3, 1) and (4, 0, −2) intensities over stages I&II indicates separate steps of lattice relaxations. The suppression up to 70% of (3, 0, −2), (3, 1, −3) intensities is the dominant feature during stage I, which indicates a de-pairing process based on symmetry argument. Meanwhile, changes over higher symmetry reflections along different planes are also observed, suggesting a three-dimensional structural changes. With the lattice parameters frozen, we conduct analyses on the initial different structural transformations directed along the two critical structural modes (pairing and twisting) on the A and B sub-lattice chains.

We find that processes in stages I&II depicted in the diffraction intensity evolution [Fig. 4(a)] can be emulated through a pathway in which M_2_^*^ (*denotes nonequilibrium, excited nature) serves as a metastable intermediate structural motif, where half of the chains are unpaired, while the other half are untwisted, as shown in Fig. 4(b) [skeleton evolution depicted in Fig. 4(c)]. In contrast, simultaneously or sequentially reducing the pairing and twisting of both sublattice chains cannot capture the key features of the experimental data (see SI for details). From the perspective of crystalline state evolution, the initial switchover to M_2_^*^ is also identified from the appearance of M_2_ (1, 1, 3) reflection (representing unitcell doubling in the basal plane) near *s* = 4 Å^−1^ as shown in Fig. 1(b). The transition into this new motif and its relaxation can be analyzed from monitoring the center-of-mass evolution of the reflection group containing the M_2_-specific reflection and nearby monoclinic (2, 2, 0) and (2, −1, 1) reflections in total diffraction. As shown in Fig. 3(d), the center-of-mass position initially moves toward strengthening the M_2_ characters, opposite to what would occur if the structure directly evolves into R, which occurs only after the second transition (stage IV) as shown in Fig. 3(a).

## Conclusion

Based on the excitation dose, wavelength, and temperature-controlled studies, the photoinduced phase transitions of VO_2_ are inspected over the dynamical signatures from multiple scattering channels, including Bragg diffraction, diffusive scattering, and coherent oscillation as a probe for acoustic waves. Several new observations were made which can be correlated with earlier pump-probe and thermodynamic measurements to address the on-going questions. Firstly, we show that the ultrafast dynamics of photoinduced phase transition in VO_2_ is density-driven and can be significantly subthermal using long wavelength photons. Secondly, we demonstrate that the phase transition involves at least two main steps with a well distinguishable intermediate state taking on a motif akin to M_2_, coupling to concerted detwisting and depairing along two different V-V sublattice chains. This symmetry change is consistent with the hole- doping picture^10^ for the initial evolution, but the extent of excess holes at the threshold level may be adequate to drive the system metallic^22^,^24^. Thirdly, we identify the unusual roles of the transient stress and their remediation through the launch of impulsive acoustic waves coupled to wavelength-dependent diffusive signatures of lattice instabilities that trigger such events. Given the decisive role of this strain relief in transitioning into the final rutile phase, one may envision using strain as a control parameter for phase transition. This may also help understand several recent accounts of unusually stable photoinduced monoclinic metal under the influence of stress^33^,^35^. Conversely, by exploiting the flexibility associated with amorphous-membrane-supported ultrafine VO_2_ nanocrystalline systems to reduce the role of the transient stress, a relatively unhindered high speed interaction-driven switching may be established.

Regarding whether the results reported here are applicable to single-crystal systems, we first draw comparisons between the temperature-mediated and photoinduced phase transition in both single-crystal and polycrystalline systems. Previous study of VO_2_ nanobeams gently placed (nearly stress-free) on a substrate showed extremely narrow transition width for both thermally and optically induced transitions (<0.2 K, <0.1 mJ/cm^2^ respectively)^27^, whereas the phase transitions in epitaxially grown VO_2_ sample can be broadened by up to tens of K due to the static strain induced by sample-substrate mismatch^34^. Meanwhile, in the nanocrystalline film reported here, the thermal transition is inhomogeneously broadened due to surface stress while experiencing lesser external stress from the substrate due to its amorphous nature. It is expected that this inhomogeneous broadening will be translated to optically driven transformation, nonetheless, we demonstrate that the difference in the thresholds observed here using two different photon wavelengths greatly exceeds the inhomogeneous widths of the respective transformations [Fig. 1(c)]. Therefore, the underlying physics of the interaction-driven transition and the emergence of transient metastable state are robust against inhomogeneity. It is interesting to point out that while the onset threshold of our experiments is found to be consistent with earlier studies using both polycrystalline and single-crystal samples^16^,^20^,^21^,^22^,^28^, the full switching threshold witnessed here is substantially lower than earlier results because of the avoidance of excessive transient stress. We expect the same processes to occur in single-crystal VO_2_ systems, particularly when steps are taken to reduce the external constraint to avoid both static and transient stress that may serve as a feedback to influence the phase transition dynamics as described earlier. Future more sensitive experiments on isolated single-crystal nanobeams under controlled stress and excitation wavelengths should provide more information on the anisotropic phonon softening tied to the phase transition and elaborate on the mechanism responsible for the decay of the metastable phase under the influence of stress.

Finally, the findings presented here should be relevant to the development of new electronic applications. Our observations suggest that carrier doping to be the key mechanism to induce the metastable M_2_* doorway state, which facilitates the ultrafast conversion of insulating M_1_ into metallic phase, even under a subthermal scenario. It is therefore reasonable to suggest that such a channel might be active through the nonequilibrium transport studies where hot carriers could be directly injected via intense current pulse or electrostatic gating. Indeed, tantalizing evidences of nonthermal insulator-metal switching^53^,^54^ operated at very high speed (~1 ns, resolution-limited) in sandwiched nanoscale VO_2_ film has recently been reported^55^. Whether or not a similar pathway is involved in the electrically driven transition as in the photo-doping scenario awaits further elucidation. It would be very interesting to explore the avenue of optically assisted insulator-metal transition using lower-energy photons taking advantage of their large penetration depth in such device implementation by leveraging the carrier-driven emergent state to circumvent thermodynamic limitations for more efficient and ultrafast switching^56^.

## Methods

### Sample synthesis

The VO_2_ thin film samples used in the experiments were prepared via pulsed laser deposition^57^,^58^ on 9 nm thick amorphous silicon (a-Si) 100 μm × 100 μm TEM membrane windows (SIMPore: US100-A09Q33). The TEM windows were first attached to a silicon substrate, which was then loaded into a custom-built vacuum chamber for the deposition of VO_2_ thin films. Before the deposition started, the background pressure was lowered to 8 × 10^−6^ Torr to remove impurity ambient gases. Upon deposition, the temperature of the sample was increased to ~550 °C, whereas the oxygen was introduced in the chamber at a constant rate of 20 standard cubic centimeters per minute (sccmm). Meanwhile, the pressure was controlled by a butterfly control valve to maintain at 15 mTorr. A krypton fluoride (KrF) excimer laser was operated at 10 Hz under a pulse energy of ~350 mJ (fluence of approximately 2 J/cm^2^) to ablate vanadium from a solid metallic target inside the chamber to grow VO_2_ films on the substrate. During deposition, the sample was constantly rotated for an even distribution of heat and material deposition. Upon completion of the deposition time, the laser was turned off and the sample went through a 30 minute annealing process under the same environment conditions. Under this process, it took ~7 minutes and 30 seconds to grow ~50 nm thick films, which was confirmed by using AFM. Using a scanning electron microscope (SEM), we can differentiate the nanosized grain composition and establish a grain size distribution to be around 31 ± 7 nm, as shown in Supplementary Fig. S1.

### Characterization

The steady-state insulator-metal transition of the as-prepared VO_2_ thin film was confirmed by temperature-dependent resistance measurement depicted in Supplementary Fig. S2(a), showing 2 orders of magnitude change in resistance across the transition temperature *T*_*C*_ ~ 340 K^6^. A dispersion of ~8 K is also identified, which we attributed to the inhomogeneity-induced broadening, rather than the intrinsic property of VO_2_, as evidenced in recent studies of single-crystal nanobeams^27^,^38^,^59^. Also shown is the structure phase transition (see Supplementary Fig. S2(b)), monitored through the electron diffraction, in which the (3, 0, −2) intensity is used as an order parameter to gauge the degree of phase transition. We note that the measured resistivity continues to change beyond the structural transition region. This trend is indicative of a continuous change in the electronic property of VO_2_ beyond *T*_*C*_, not correlated with the structural phase transition^38^,^59^.

### Ultrafast electron diffraction

The ultrafast electron diffraction experiments were conducted using the ultrafast electron crystallography (UEC) and the ultrafast electron microdiffraction (UEmD) systems. The low temperature studies were performed in the UEC system where the sample goniometer can be cooled to 20 K^5^. Temperature was measured with 0.01 K precision at the back of sample holder, which was in a solid thermal contact with the sample grid, which was clamped into a copper counter sink on the sample holder. The probe size was controlled via adjusting a condenser lens in front of the sample to 30 μm in full-width-at-half-maximum (FWHM) in this experiment ensuring the electron beam was fully contained near the center of the VO_2_ film deposited on the 100 um × 100 um TEM membrane window. The pump pulse was generated by an optical parametric amplifier driven by a 45 fs, 800 nm amplified laser system (Spectra Physics, Ti: Sapphire, Regenerative Amplification), and can be tuned from 200 nm to 3000 nm in wavelength. In this experiment, primarily 800 nm and 2000 nm wavelength were used. The size of the pump beam at different wavelength was adjusted by varying the position of a focusing lens. Its size along x direction (see Supplementary Fig. S3(a)) was directly measured via knife-edge measurement *in situ*, while the aspect ratio of the pump laser spot was determined *in situ* using laser-electron cross-correlation approach (see SI). Part of the incident laser beam was also split off by a beam splitter before entering the diffraction chamber, which was monitored with a beam profiler to cross check the size and intensity of the beam during the experiment. We set the pump size to be ~400 μm in FWHM, which was significantly larger than the probe size to ensure that within the probed area the sample was homogeneously illuminated by the pump laser. The room temperature studies were also conducted using the UEmD system where the beam energy can be adjusted up to 100 keV. We determine the normalized diffraction intensity changes do not vary strongly over the beam energy. We kept the repetition rate to be ≤500 Hz to ensure there was no residual heating effect that would change the initial state. This is verified by *in situ* monitoring the diffraction patterns produced at negative timeframes to be consistent with that obtained from the sample without optical pumping, and the threshold dose at a given temperature is independent of the exposure time (see Supplementary Fig. S4).

## Additional Information

**How to cite this article**: Tao, Z. *et al*. The nature of photoinduced phase transition and metastable states in vanadium dioxide. *Sci. Rep.* **6**, 38514; doi: 10.1038/srep38514 (2016).

**Publisher's note:** Springer Nature remains neutral with regard to jurisdictional claims in published maps and institutional affiliations.

## Supplementary Material

Supplementary Information
