# Supplementary material for: The nature of photoinduced phase transition and metastable states in vanadium dioxide
Source: Sci Rep. 2016 Dec 16;6:38514. doi: 10.1038/srep38514 (PMC5159834; doi:10.1038/srep38514)
Supplement: Supplementary Information [file srep38514-s1.pdf]

# Supplementary Information

# The nature of photoinduced phase transition and metastable states in vanadium dioxide

Zhensheng Tao[1], Faran Zhou[1], Tzong-Ru T. Han[1], David Torres[2], Tongyu Wang[2], Nelson Sepulveda[2], Kiseok Chang[1], Margaret Young[3], Richard R. Lunt[3,1], Chong-Yu Ruan[1]*

[1] Department of Physics and Astronomy, Michigan State University, East Lansing, Michigan 48824.
[2] Department of Electrical and Computer Engineering, Michigan State University, East Lansing, Michigan 48824.
[3] Department of Chemical Engineering and Materials Science, Michigan State University, East Lansing, Michigan 48824.

*Table of contents*

**I. Sample Synthesis and Characterization**

**II. Experimental Setup**

**III. Calibration of Optical Constants**

**IV. Determination of Energy and Optical Dose**

**V. Structure Analysis**

---

*For correspondence: ruan@pa.msu.edu

# I. Sample Synthesis and Characterization

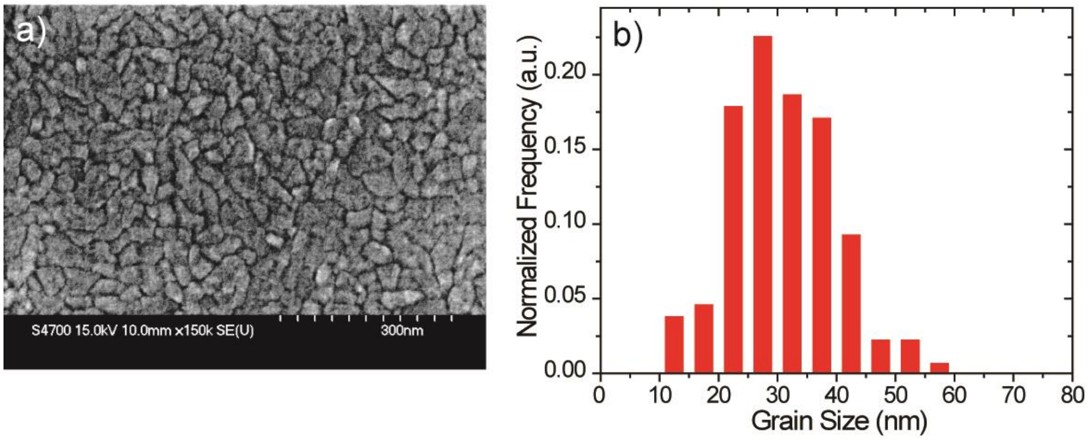


**Figure S1: Sample Geometry.** a) SEM image of the $VO_2$ thin film. b) Histogram showing the distribution of the nano-grain size in the $VO_2$ thin film determined from SEM images. The mean grain size is ~31 nm, with a 1σ inhomogeneous broadening of ~ 7nm.

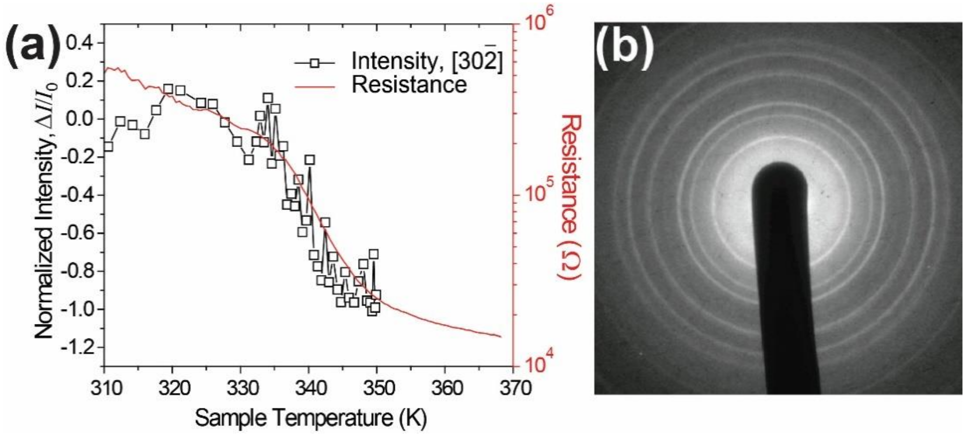


**Figure S2: Characterization of the $VO_2$ thin.** (a) Thermally induced phase transitions in the thin film. The open squares show the intensity change of the Bragg reflection (30 $\bar{2}$ ), whose intensity decay represents structural transformation of $VO_2$. The red solid line represents the insulator-metal transition, which is measured on the substrate area via the four-probe method. The critical temperatures of structural and electronic transitions are near ~339 K, over a transition temperature window of ± 8 K. (b) Room-temperature ground-state electron diffraction pattern obtained using the ultrafast electron diffraction setup.

# II. Experimental Setup

The *in situ* knife-edge measurements only allow us to measure the pump-laser spot size along *x* direction, while we determine the size in the other direction (*y*) by laser-electron cross-correlation approach, as illustrated in Fig. S3(a). In this measurement, we move the pump-laser spot on the sample and monitor the changes of electron diffraction patterns of the sample at a time delay ~+100 ps, which gives us the cross-correlation response between the IR pump laser and the electron beam.

Because the electron beam is focused to ~30 μm on the sample that is much smaller than the pump laser spot size, the cross-correlation measurement can give a reliable measurement of the pump-laser spot size. The intensity of selected Bragg reflection as a function of the displacement of pump laser spot along *x* direction ($\Delta x_{pump}$) and along *y* direction ($\Delta y_{pump}$) for both pump wavelengths (800 nm and 2000 nm) is plotted in Fig. S3(b). We note that the displacement values shown in Fig. S3(b) correspond to the internal reading of the motor which we used to control the moving optics, so they do not directly give the real-space size of the laser pump spot. With proper calibrations, the measurements can provide us a measurement of the aspect ratio of the pump-laser spot for both wavelengths. The estimated error in pump-size is ~17% for 800nm and ~25% for 2000 nm pump, which is determined by both knife-edge measurements and the laser-electron cross-correlation measurements.

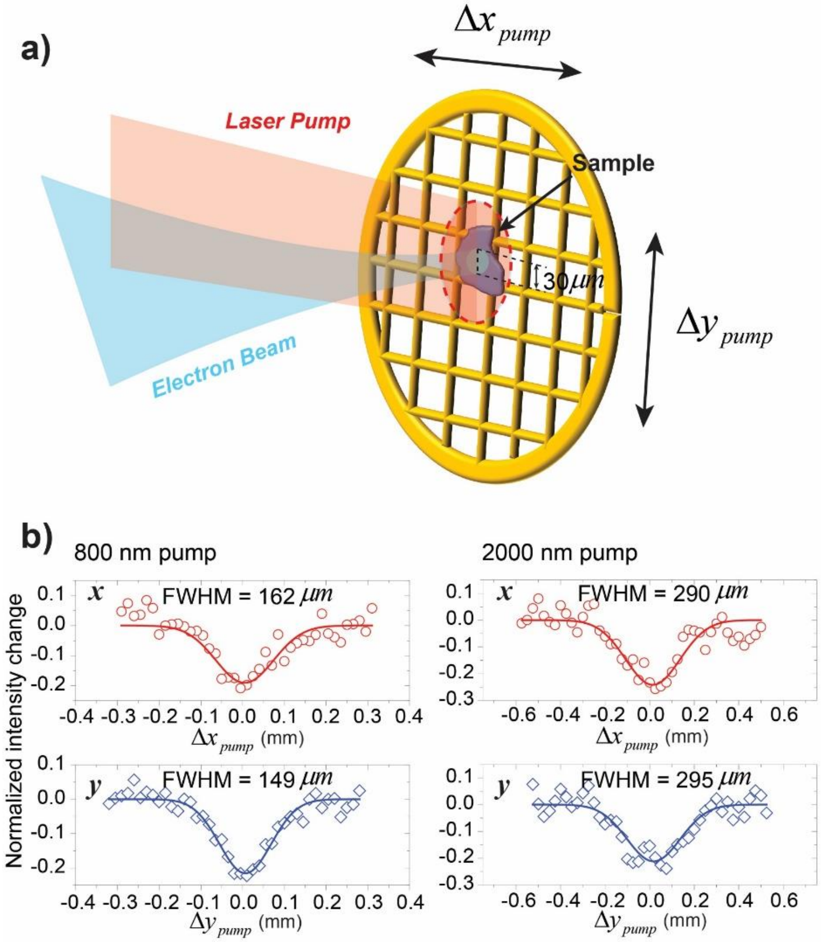


**Figure. S3: Laser-electron cross-correlation measurements.** a) Experimental setup of the measurement. The electron beam is focused to ~30 μm on the sample. The laser spot is ~400 μm in size. The laser spot is moved by a motor controlled optics with well-defined steps and the variation of the diffraction pattern is measured after sample. b) The intensity of the selected Bragg reflection is plotted as a function of the displacement of the laser spot on sample along *x* and *y* directions. Note that the internal reading of the motor that is linearly related to the real-space displacement is used in the plots.

The repetition-rate dependent study has been conducted to verify the full recovery of the samples. Fig. S4 show the results conducted at the base temperature $T_B$=290K. When the repetition rate is set at 1 kHz (1ms between pump-probe cycles), the intensity of $(30\bar{2})$ peak before zero-of-time (-100 ps) decreases rapidly when pump laser fluence increases. The transition fluence for the intensity at 150 ps is ~3.5 mJ/cm$^2$, much lower than the results obtained at the repetition rates of 500 Hz and 200 Hz.  So, the negative-time state of $VO_2$ has an increased temperature than $T_B$ in the 1kHz repetition rate experiments due to the thermal build-up between the cycles, resulting in a lower than expected threshold energy. In contrast, at 500 Hz repetition rate, the intensity change at -100 ps is close to zero compared to pump-blocked intensity, even when laser fluence is increased to very high level. By further reducing repetition rate to 200 Hz, we observe the results of fluence scan are consistent with 500 Hz results, which means that at 500 Hz (2ms between cycles) $VO_2$ samples can fully recover to its ground state as without applying the laser pulses. So, the full thermal recover time of our sample under ultrahigh vacuum is between 1ms to 2ms. By operating experiment at ≤500 Hz, we can eliminate the effects of thermal build-up at the negative times.

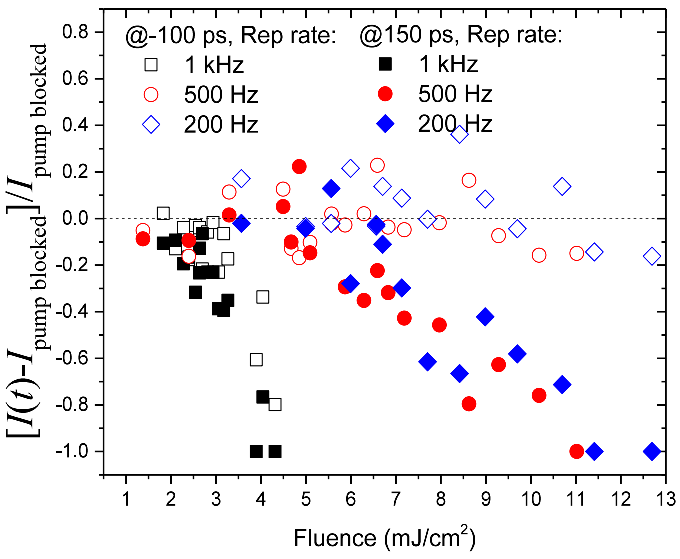


**Figure S4: Repetition rate dependent study.** The intensity change of the Bragg reflection $(30\bar{2})$ at negative (-100 ps, hollow symbols) and positive (+150 ps, solid symbols) times over the fluence ranges covering $M_1$-to-R transition were determined at repetition rate of 200, 500 Hz, and 1kHz.  The negative time signals obtained at 1kHz show fluence dependence, indicating that the sample is not fully recovered. In contrast, the experiments conducted at 200 and 500 Hz show no fluence dependence in the negative time frames and have a similar transition curve, indicating that the $VO_2$ samples pumped at these two repetition rate are fully reversible.

## III. Calibration of Optical Constants

The optical constants of the employed $VO_2$ samples were determined based on the broadband optical reflectance and transmittance measurements on an as-grown 50 nm $VO_2$ films, deposited on a 45 nm a-Si layer sputtered on a quartz substrate.  The optical transfer matrix[60] $\hat{T}$

was constructed to formulate the correlation between optical transmission and reflection in each layer:

$$\begin{Bmatrix} A_m^+ \\ A_m^- \end{Bmatrix} = \hat{T} \begin{Bmatrix} A_{m-1}^+ \\ A_{m-1}^- \end{Bmatrix}. \qquad \textbf{(S1)}$$

Here, we denote $A_m^+$ as the amplitude of the wave field traveling in the direction of the incidence in the $m^{th}$ layer and $A_m^-$ as that of the reflected wave, as shown in the Fig. S5(a). The transfer matrix of the $m^{th}$ layer in the film is directly related to its optical constants by

$$\hat{T}_m = \frac{1}{t_m} \begin{Bmatrix} e^{i\chi_{m-1}} & r_m e^{-i\chi_{m-1}} \\ r_m e^{i\chi_{m-1}} & e^{-i\chi_{m-1}} \end{Bmatrix}. \qquad \textbf{(S2)}$$

Here, $t_m = 2n_m/(n_m + n_{m-1})$, $r_m = (n_m - n_{m-1})/(n_m + n_{m-1})$ and $\chi_m = n_m k_0 a_m$, where $n_m$ is the refractive index of the $m^{th}$ layer, $k_0 = 2\pi/\lambda$ is the wave-vector of the light in vacuum and $a_m$ is the thickness of the $m^{th}$ layer. To describe a lossy material with absorption and loss, the complex refractive index $n = n^0 + i\kappa$ is implemented in the elements of transfer matrix, where $n^0$ and $\kappa$ are the real and imaginary parts of the refractive index, respectively. $\kappa$ is frequently referred to as the extinction coefficient, which is related to the material's optical penetration depth $\delta$ with $\delta = \lambda/(4\pi\kappa)$.

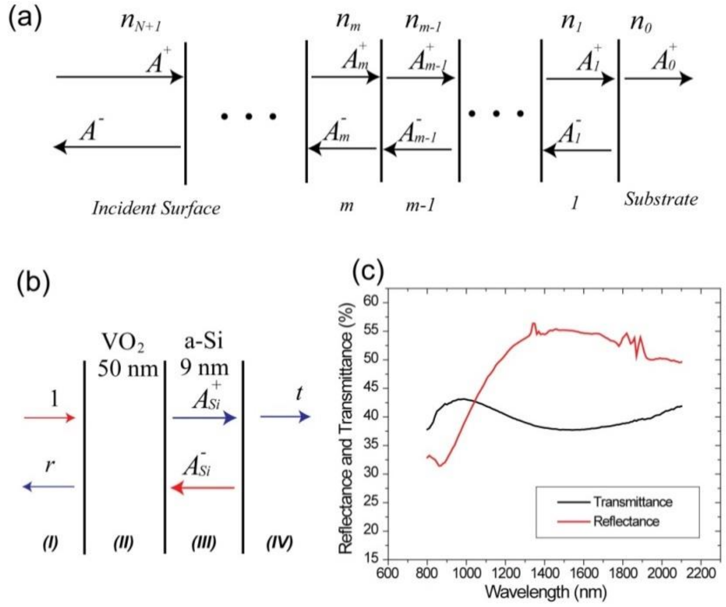


**Figure S5: Schematic representation of the optical transfer matrix in multilayers and the experimental results for $VO_2$-a-Si composite film.** a) General multilayer thin-film system with $N$ layers of films. $A_m^+$ represents the amplitude of the incident light wave field, while $A_m^-$ is that of the reflected wave field at the $m^{th}$ layer. $n_m = n_m^0 + i\kappa_m$ is the complex refractive index of the $m^{th}$ layer. b) The wave fields in each region of $VO_2$-a-Si composite film used for calibration. c) The measured reflectance and transmittance on the calibration sample.

For the specific multilayer $VO_2$ film (Fig. S5(b)) the overall reflectance *R* and transmittance *T* measured experimentally can be linked to $\hat{T}_{VO2}$ and $\hat{T}_{a-Si}$, representing the transfer matrix of the $VO_2$ and a-Si films:

$$\begin{Bmatrix} 1 \\ r \end{Bmatrix} = \hat{T}_{VO2}\hat{T}_{a-Si}\begin{Bmatrix} t \\ 0 \end{Bmatrix}, \qquad \textbf{(S3)}$$

where *r* and *t* represent reflected and transmitted wave field in the regions I and IV, determining *R (r*r)* and *T (t*t)*. The measured wavelength-dependent optical transmittance and reflectance are depicted in Fig. S5(c). The optical constants of the a-Si thin film are pre-calibrated via the similar transmittance and reflectance measurements to construct $\hat{T}_{a-Si}$. The refractive index $n^0$ and κ of $VO_2$ are thus determined by fitting the reflectance and transmittance data using Eqn. S3. The $n^0$ and $\delta$ of $VO_2$ obtained from fitting are listed in the Supplementary Table 1. For comparison, the optical constants for bulk $VO_2$ deduced from literature[15,61] are listed in the Supplementary Table 2.

| Wavelength (nm) | $n^0$ | $\delta$ (nm) | $\gamma$ | $\gamma'$ |
|---|---|---|---|---|
| 800 | 2.85 | 100 | 0.213 | 0.296 |
| 1319 | 3.2 | 300 | 0.145 | 0.111 |
| 2000 | 3.21 | 1061 | 0.079 | 0.033 |

**Supplementary Table 1: Optical refractive index and penetration depth of $VO_2$ film at three different IR wavelengths.** The real part of index refraction $n^0$ and the penetration depth $\delta$ are deduced from data presented in Fig. S5(c) using the transfer matrix method. Also listed are the absorption ratio $\gamma$ of the 50nm $VO_2$ film used in the ultrafast measurements, which are determined by applying the calibrated optical transfer matrix. The absorption ratio $\gamma'$, calculated without considering the interference effect is also listed for comparison.

| Wavelength (nm) | $n^0$ | $\kappa$ | $\delta$ (nm) | $\gamma'$ |
|---|---|---|---|---|
| 800 | 2.88 | 0.42 | 152 | 0.212 |
| 1319 | 3.16 | 0.39 | 269 | 0.123 |
| 2000 | 3.25 | 0.13 | 1224 | 0.028 |

**Supplementary Table 2: Optical refractive index and penetration depth of $VO_2$ at three different IR wavelengths deduced from literature values.** The listed absorption ratio $\gamma'$ is calculated without considering the interference effect for comparison.

We calculate the absorption ratio γ, defined here as the ratio between the net absorbed fluence ($F_{abs}$) by the $VO_2$ film and the applied fluence *F*, namely $\gamma = F_{abs}/F$. Assuming the incident light-wave amplitude to be unitary, the absorption ratio of the 50 nm $VO_2$ film is directly given by

$$\gamma = \left(1 + n_{Si}A_{Si}^{-} \cdot A_{Si}^{-*}\right) - \left(r \cdot r^{*} + n_{Si}A_{Si}^{+} \cdot A_{Si}^{+*}\right), \qquad \textbf{(S4)}$$

where $n_{Si}$ is the refractive index of the silicon film, $A_{Si}^{-}$ ($A_{Si}^{+}$) and $A_{Si}^{-*}$ ($A_{Si}^{+*}$) are the amplitude of the reflected (incident) wave field in the a-Si layer and its complex conjugate, respectively. These parameters along with the regions they are defined can be found in Fig. S5(b). The calculation takes into account the difference in the supporting a-Si membranes. In the ultrafast electron diffraction experiment, the a-Si membrane is reduced to 9 nm to ensure transparency for

transmission electron diffraction studies. The reported γ is determined based on the transfer matrix using the measured optical constants applied to the 9nm a-Si substrate. Nonetheless, it was found that up to 98% of the absorbed optical energy is absorbed by the 50 nm $VO_2$ film in the optical wavelength ranging from 800 nm to 2000 nm in the calibration experiment, hence the absorption by the 9 nm a-Si thin film is in fact negligible.

To compare with other results reported in the literature, it's interesting to see the difference in γ due to the optical interference in the thin film geometry. Without considering interference, $R=\frac{(n^0-1)^2+\kappa^2}{(n^0+1)^2+\kappa^2}$ and $\delta=\frac{\lambda}{4\pi\kappa}$ , and the absorption ratio γ' in such a scenario is $(1-R)\left[1-\exp\left(-\frac{t}{\delta}\right)\right]$. Table S1 shows the comparison between γ and γ', for the 50nm $VO_2$ films. The main difference occurs for 2000nm, where the net absorption is reduced to half if the interference effect were to be ignored. Including such an effect, γ differs by nearly 300% between 800 nm and 2000 nm. However, if the optical interference were to be ignored, the difference would further increase. Such suppression of optical absorption in mid-IR is well linked to the diminishing density of states near the optical gap edge.

## IV. Determination of Energy and Optical Dose

With Table S1, we can now calculate the absorbed energy density $\Delta H$ :

$$\Delta H=\frac{\gamma F}{a_{VO_2}}, \tag{S5}$$

where $a_{VO_2}$ is the thickness of the $VO_2$ film (50 nm). For consideration of optical doping effect, we calculate the optically generated electron and hole density, which is equal to the absorbed photon density $n_\lambda$:

$$n_\lambda=\frac{\Delta H}{E_\lambda}. \tag{S6}$$

To quantitatively compare with the optically induced phase transitions, as shown in Fig. 2(a), we calculate the absorbed thermal energy density in the temperature-induced phase transition at temperature $T$, based on integrating the heat capacity $C_v$ [42] and latent heat $L_0$ of $VO_2$ from the initial crystal temperature $T_B$ to $T$:

$$\Delta H(T)=\int_{T_i}^{T} C_v \mathrm{d}T+L(T). \tag{S7}$$

The latent heat in single crystal $VO_2$, $L_0$, has been determined to be $235\times10^6$ $J/m^3$ [42], or 1.47 $eV/nm^3$.

## V. Structure Analysis

The local bonding changes associated with optically induced $M_1$ to R transition can be inspected based on the time-dependent radial distribution function *G(r; t)*, obtained via a Fourier transform of the normalized structure function *sM(s)* presented in Fig. 1(b)[62]. Fig. S6(b) depicts the difference radial distribution function *ΔG(r;t)*, obtained by taking the difference between *G(r; t)* and *G(r; -2ps)*. *ΔG(r;t )*can be used to identify the formation of new bonds and depletion of old bonds. In transition from the $M_1$ state to the R state, the correlation density at the location of $r_R$ (2.85Å) will increase, whereas the correlation density near $r_S$ and $r_L$ will decrease in the nearest V-V bonding region – see Fig. S6(a). Similar trend will also occur in the 2nd nearest neighbor region around 3.52Å, representing the closest V-V bonding between the two sublattice chains, as indicated in Fig. S6(a). These phenomena are evidenced in Fig. S6(b). Whereas the radial distribution function analysis is informative of local bonding changes, for understanding the symmetry-related structural modes directly coupled to electronic excitations, it is more straightforward to inspect the integrated intensity of symmetry-related Bragg reflections.

The atomic movements within the unit cell lead to effects of constructive (intensity increase) or destructive (intensity decrease) interferences of the scattered electron waves, directly contributing the observed diffraction intensities. These changes can be determined by the change of the structure factor for a given direction and zone axis. Specifically, the structural factor $F(\theta_{hkl})$ is the sum of the scattered electron waves from every atom within the unit cell:

$$F(\theta_{hkl}) = \sum_i f_i(\theta_{hkl}) e^{2\pi i(hx_i + ky_i + lz_i)}, \qquad \textbf{(S8)}$$

where $\theta_{hkl}$ is the scattering angle associated with the Miller indices (*hkl*), $f_i$ and $(x_i; y_i; z_i)$ are the form factor and the fractional coordinates, respectively, of the i-th atom in the unit cell. The intensity of the Bragg peak $I_{hkl}$ is proportional to the square of $F(\theta_{hkl})$:

$$I_{hkl} = |F(\theta_{hkl})|^2. \qquad \textbf{(S9)}$$

Phase transition from the R to the $M_1$ state can be well described by two symmetry-breaking structural modes, namely the pairing of V-V bonding along the c-axis, and the twisting of V-V bonding involving displacements of V atoms towards ***ab*** plane (see Fig. S6(b)). These two independent structural modes can manifest in diffraction intensity over two different sets of Bragg reflections. For tracking the pairing status, we can resort to $(3\ 0\ \bar{2})_{M1}$ and $(3\ 1\ \bar{3})_{M1}$, which arise only after the lattice dimerizes along the **c**-axis. In contrast, we can also identify $(2\ 2\ 0)_{M1}$ and $(2\ 3\ \bar{1})_{M1}$, which carry strong contributions from twisting mode. The symmetry recovery from $M_1$-to-R transition will diminish the $(3\ 0\ \bar{2})$ and $(3\ 1\ \bar{3})$ intensities and increase the (2 2 0) and $(2\ 3\ \bar{1})$ intensities. This can occur without changing the octahedral motif, namely the unit cell constant does not instantaneously change over the ultrafast timescale[16].

The atomic displacement vectors associated with the two structural modes are built on the fractional atomic coordinates based on a fixed unit cell. In the models, the O atoms are frozen during the processes of depairing and detwisting, based on our current understanding of electronically driven structure phase transition between $M_1$ and R[8]. We also keep the volume constant as discussed, namely the unit cell constants are also frozen, only the fractional atomic positions are adjusted. From $M_1$ to R, the unit cell size is reduced by a factor of two exactly. This is in contrast to thermally induced transition, where a lattice strain is transferred from the $M_1$

structure to the R structure[40,63,64], which leads the following ratios: $\frac{c_{M1}\sin(\beta_{M1})}{c_R} = 1.0159$, $\frac{-c_{M1}\cos(\beta_{M1})}{a_R} = 0.9941$ and $\frac{a_{M1}}{2c_R} = 1.0070$, to deviate from unity, where $(a_{M1}, b_{M1}, c_{M1})$ and $(a_R, b_R, c_R)$ are the lattice constants for $M_1$ and R phases respectively and $\beta_{M1}$ is the interaxial angle between $\vec{a}_{M1}$ and $\vec{c}_{M1}$. Here, we set all three ratios to unity throughout the phase transition, justified by the fact that changes in unit cell distances will only affect the position of the Bragg reflections, not the integrated intensity.

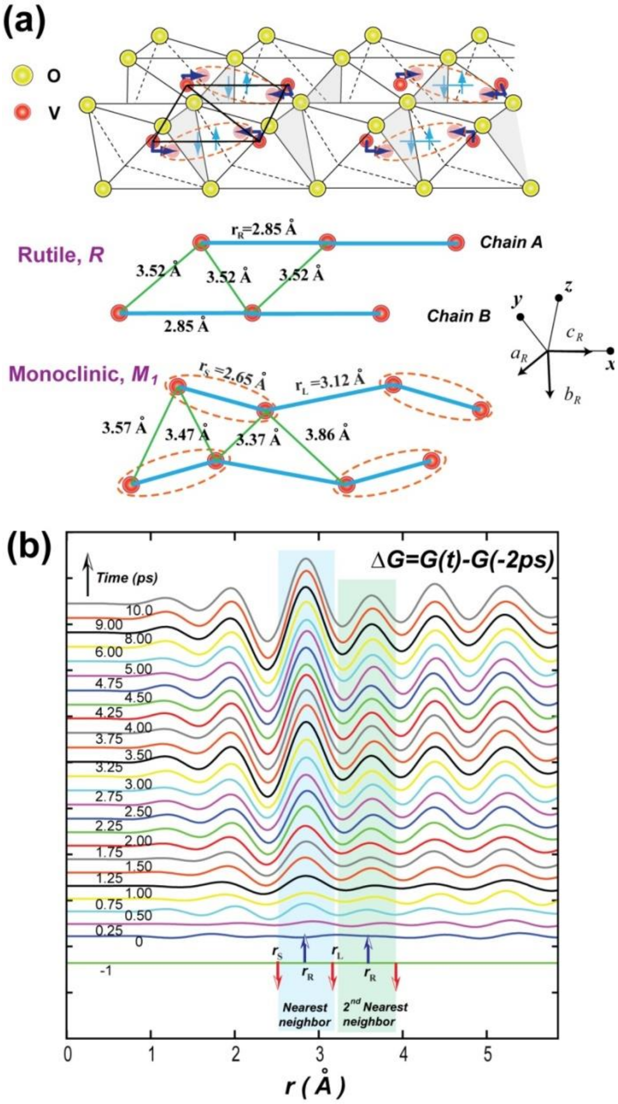


**Figure S6: Nearest neighbor bonding in $VO_2$.** (a) The 5nearest and 2[nd] neareast V-V distances in the $M_1$ and the R states. (b) The radial distribution function *G(r)* analysis showing the changes in local bonding from photo-induced $M_1$-to-R transition under mid-IR excitation. The difference curves *ΔG(t)* is taken using *G(r)* at -2 ps as the reference.

In simulating the scattering intensity, we used the Mott-Bethe formula to convert the X-ray form factors to the electron ones[65]. The crystal unit cell is based on x-ray crystallography results of $M_1$[40]. For fitting the dynamics as shown in the diffraction intensity evolution (Fig. S7a), we employed three different dynamical models pathways (see Fig. S7b). Model A considers continuous movements of bond distances and angles from $M_1$ to R as a result of superposing the two structures, which emulates a thermal transition where the intermediate states are mixtures of $M_1$ and R domains prior to establishing a uniform R state. Model B considers a simple two-step transformation from two separate modes with the step (i) predominately conducting de-pairing along $\mathbf{c_R}$, which is followed by the step (ii) changing course to de-twisting. These movements take place simultaneously on A and B chains. Model C considers a metastable $M_2$ separating the two steps. In Model C, sublattices undergo separate de-pairing and de-twisting towards untwisted A and unpaired B chains at the $M_2$ stage. The detailed structural displacement steps associated with the three models are depicted in Fig. S7(b) along with the corresponding simulated intensity changes for the 5 Bragg reflections as depicted in Fig. S7(a). The overall velocity of the movements was obtained by fitting $(3\ 0\ \bar{2})_{M1}$ and $(3\ 1\ \bar{3})_{M1}$ simulated intensities to the data and the intensity changes of the Bragg reflections $(2\ 2\ 0)_{M1}$, $(4\ 0\ \bar{2})_{M1}$ and $(2\ 3\ \bar{1})_{M1}$ were generated accordingly. The results are presented in Fig. S7(b). From the key features of evolutions, we

clearly rule out Model A for lacking any visible step-wise changes in all the intensities. Model B shows steps in $(220)_{M1}$, $(23\bar{1})_{M1}$ (as expected), but not in $[40\bar{2}]_{M1}$, which rises unceasingly with a disproportionally higher intensity than those of $(220)_{M1}$, $(23\bar{1})_{M1}$ within the step (i), which is inconsistent with the data. Mode C agrees best with the data, reproducing all the key features.

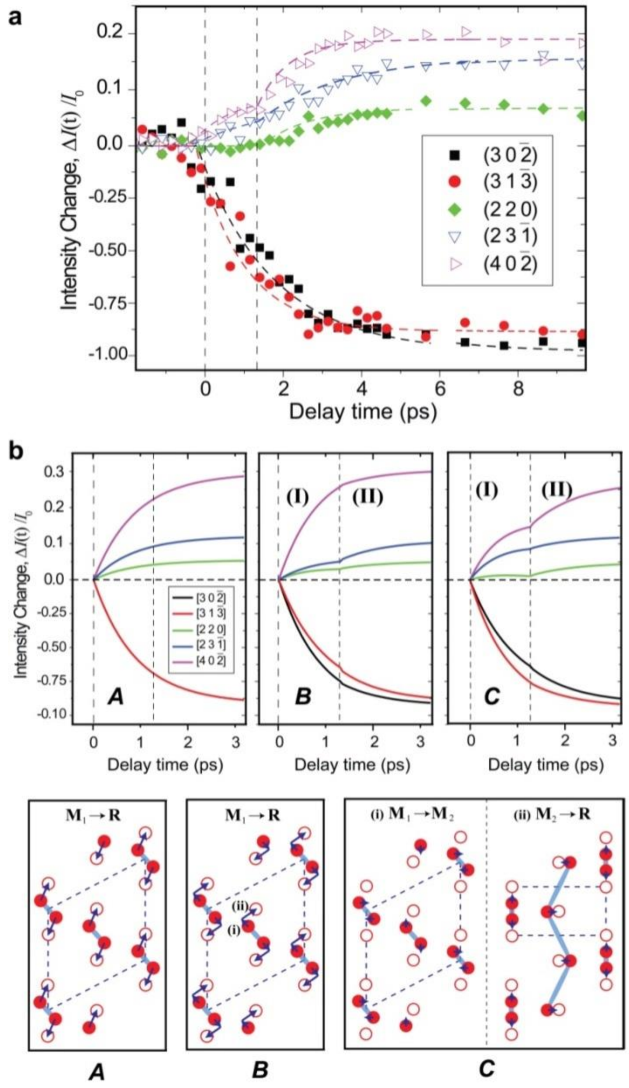


**Figure S7: Ultrafast atomic transformation pathway of $VO_2$ under photodoping. a**, The intensity evolution of the Bragg reflections during structural phase transition from $M_1$ to R state. The dashed lines are drawn as a guide to the eyes. **b**, Intensity changes of the corresponding Bragg reflections simulated by displacing the V atoms as shown in the panels below using three different structural models for phase transitions.

The Model C agrees well with the experimental results despite of the fact that the movements of individual atoms are highly constrained to only two different structural modes. We note, in reaching this conclusion we have examined the influence of the scale factors of the two structural modes in fitting the experimental results. Using the same scale factor for both structural modes, we found the changes associated with $(2\ 2\ 0)_{M1}$, $(4\ 0\ \bar{2})_{M1}$, and $(2\ 3\ \bar{1})_{M1}$ reflections, which are directly sensitive to the twisting mode, are greater than the experimental values. Simply reducing the initial detwisting angle by 50%, the agreement is drastically improved. This may suggest that the initial monoclinic state under the surface strain has a reduced lateral distortion, whereas the dimerized V-V bonding distance remains near that of pristine $M_1$ state, putting such a strained initial state to be between the pristine $M_1$ and $M_2$ states. More sophisticated modeling using separate scaling factor to model the amplitude of the two structural modes may capture more details of changes. The model presented here represents the simplest self-consistent model, capturing the essential features of the experiments. We note that in various strain-controlled $VO_2$ nanobeam experiments, the intermediate monoclinic insulating states, namely T or $M_2$, may become meta-stable[38,59,66]. Such observations could provide the basis for understanding the ground state properties of $VO_2$ nanocrystals. Nonetheless, our results stand on a transient meta-stable state well distinguished in the dynamics on the 1-2 ps timescales, which can only be understood as being driven by electronic excitations, rather than strain or temperature.

**References**


1. Nasu, K. *Photoinduced phase transitions*. (World Scientific Pub. Co. Inc., 2004).
2. Fausti, D. *et al.* Light-induced superconductivity in a stripe-ordered cuprate. *Science* **331,** 189–191 (2011).
3. Stojchevska, L. *et al.* Ultrafast switching to a stable hidden quantum state in an electronic crystal. *Science* **344,** 177–180 (2014).
4. Ichikawa, H. *et al.* Transient photoinduced 'hidden' phase in a manganite. *Nat. Mater.* **10,** 101–105 (2011).
5. Han, T.-R. T. *et al.* Exploration of metastability and hidden phases in correlated electron crystals visualized by femtosecond optical doping and electron crystallography. *Sci. Adv.* **1,** e1400173 (2015).
6. Morin, F. J. Oxides which show a metal-to-insulator transition at the neel temperature. *Phys. Rev. Lett.* **3,** 34–36 (1959).
7. Whittaker, L., Patridge, C. J. & Banerjee, S. Microscopic and nanoscale perspective of the metal-insulator phase transitions of $VO_2$: Some new twists to an old tale. *J. Phys. Chem. Lett.* **2,** 745–758 (2011).
8. Eyert, V. The metal-insulator transitions of $VO_2$ : A band theoretical approach. *Ann. Phys.* **11,** 650–702 (2002).
9. Wu, Y. *et al.* Depressed transition temperature of $W_xV_{1-x}O_2$: mechanistic insights from the X-ray absorption fine structure (XAFS) spectroscopy. *Phys. Chem. Chem. Phys.* **16,** 17705 (2014).
10. Pouget, J. P. *et al.* Dimerization of a linear Heisenberg chain in the insulating phases of $V_{1-x}Cr_xO_2$. *Phys. Rev. B* **10,** 1801–1815 (1974).
11. Biermann, S., Poteryaev, A., Lichtenstein, A. I. & Georges, A. Dynamical singlets and correlation-assisted peierls transition in $VO_2$. *Phys. Rev. Lett.* **94,** 26404 (2005).
12. Weber, C. *et al.* Vanadium dioxide: A peierls-mott insulator stable against disorder. *Phys. Rev. Lett.* **108,** 1–5 (2012).
13. Zylbersztejn, A. & Mott, N. F. Metal-insulator transition in vanadium dioxide. *Phys. Rev. B* **11,** 4383–4395 (1975).
14. Goodenough, J. B. The two components of the crystallographic transition in $VO_2$. *J. Solid State Chem.* **3,** 490–500 (1971).
15. Cavalleri, A., Dekorsy, T., Chong, H. H. W., Kieffer, J. C. & Schoenlein, R. W. Evidence for a structurally-driven insulator-to-metal transition in $VO_2$: A view from the ultrafast timescale. *Phys. Rev. B* **70,** 161102(R) (2004).
16. Baum, P., Yang, D.-S. & Zewail, A. H. 4D visualization of transitional structures in phase

transformations by electron diffraction. *Science* **318,** 788–792 (2007).
17. Cavalleri, A. *et al.* Femtosecond structural dynamics in $VO_2$ during an ultrafast solid-solid phase transition. *Phys. Rev. Lett.* **87,** 237401 (2001).
18. Cocker, T. L. *et al.* Phase diagram of the ultrafast photoinduced insulator-metal transition in vanadium dioxide. *Phys. Rev. B* **85,** 155120 (2012).
19. Hilton, D. J. *et al.* Enhanced photosusceptibility near $T_c$ for the light-induced insulator-to-metal phase transition in vanadium dioxide. *Phys. Rev. Lett.* **99,** 226401 (2007).
20. Kübler, C. *et al.* Coherent structural dynamics and electronic correlations during an ultrafast insulator-to-metal phase transition in $VO_2$. *Phys. Rev. Lett.* **99,** 116401 (2007).
21. Pashkin, A. *et al.* Ultrafast insulator-metal phase transition in $VO_2$ studied by multiterahertz spectroscopy. *Phys. Rev. B* **83,** 1–9 (2011).
22. Wegkamp, D. *et al.* Instantaneous band gap collapse in photoexcited monoclinic $VO_2$ due to photocarrier doping. *Phys. Rev. Lett.* **113,** 2–6 (2014).
23. Becker, M. F. *et al.* Femtosecond laser excitation of the semiconductor-metal phase transition in $VO_2$. *Appl. Phys. Lett.* **65,** 1507–1509 (1994).
24. Rini, M. *et al.* Optical switching in $VO_2$ films by below-gap excitation. *Appl. Phys. Lett.* **92,** 181904 (2008).
25. Cavalleri, A. *et al.* Band-selective measurements of electron dynamics in $VO_2$ using femtosecond near-edge X-ray absorption. *Phys. Rev. Lett.* **95,** 67405 (2005).
26. Tao, Z., Han, T. R. T. & Ruan, C. Y. Anisotropic electron-phonon coupling investigated by ultrafast electron crystallography: Three-temperature model. *Phys. Rev. B* **87,** 235124 (2013).
27. Tao, Z. *et al.* Decoupling of structural and electronic phase transitions in $VO_2$. *Phys. Rev. Lett.* **109,** 166406 (2012).
28. Morrison, V. R. *et al.* A photoinduced metal-like phase of monoclinic $VO_2$ revealed by ultrafast electron diffraction. *Science* **346,** 445–448 (2014).
29. Zeiger, H. J. *et al.* Theory for displacive excitation of coherent phonons. *Phys. Rev. B* **45,** 768–778 (1992).
30. Wall, S. *et al.* Ultrafast changes in lattice symmetry probed by coherent phonons. *Nat. Commun.* **3,** 721 (2012).
31. O'Callahan, B. T. *et al.* Inhomogeneity of the ultrafast insulator-to-metal transition dynamics of $VO_2$. *Nat. Commun.* **6,** 6849 (2015).
32. Kim, H.-T. *et al.* Monoclinic and correlated metal phase in $VO_2$ as evidence of the mott transition: Coherent phonon analysis. *Phys. Rev. Lett.* **97,** 266401 (2006).
33. Laverock, J. *et al.* Direct observation of decoupled structural and electronic transitions and an

ambient pressure monocliniclike metallic phase of $VO_2$. *Phys. Rev. Lett.* **113,** 216402 (2014).
34. Liu, M. K. *et al.* Anisotropic electronic state via spontaneous phase separation in strained vanadium dioxide films. *Phys. Rev. Lett.* **111,** 96602 (2013).
35. Hsieh, W.-P. *et al.* Evidence for photo-induced monoclinic metallic $VO_2$ under high pressure. *Appl. Phys. Lett.* **104,** 21917 (2014).
36. Sciaini, G. & Miller, R. J. D. Femtosecond electron diffraction: heralding the era of atomically resolved dynamics. *Reports Prog. Phys.* **74,** 96101 (2011).
37. Hawkes, P. W. *Time Resolved Electron Diffraction: For Chemistry, Biology And Material Science, Volume 184 (Advances in Imaging and Electron Physics)*. (Academic Press, 2014).
38. Wei, J., Wang, Z., Chen, W. & Cobden, D. H. New aspects of the metal-insulator transition in single-domain vanadium dioxide nanobeams. *Nat. Nanotechnol.* **4,** 420–424 (2009).
39. Marezio, M., McWhan, D. B., Remeika, J. P. & Dernier, P. D. Structural aspects of the metal-insulator transitions in Cr-doped $VO_2$. *Phys. Rev. B* **5,** 2541–2551 (1972).
40. Longo, J. M. & Kierkegaard, P. A refinement of the structure of $VO_2$. *Acta Chem. Scand.* **24,** 420–426 (1970).
41. Kucharczyk, D. & Niklewski, T. Accurate X-ray determination of the lattice parameters and the thermal expansion coefficients of $VO_2$ near the transition temperature. *J. Appl. Crystallogr.* **12,** 370–373 (1979).
42. Berglund, C. N. & Guggenheim, H. J. Electronic properties of $VO_2$ near the semiconductor-metal transition. *Phys. Rev.* **185,** 1022–1033 (1969).
43. Budai, J. D. *et al.* Metallization of vanadium dioxide driven by large phonon entropy. *Nature* **515,** 535–539 (2014).
44. Koethe, T. C. *et al.* Transfer of spectral weight and symmetry across the metal-insulator transition in $VO_2$. *Phys. Rev. Lett.* **97,** 116402 (2006).
45. Verleur, H. W., Barker, A. S. & Berglund, C. N. Optical properties of $VO_2$ between 0.25 and 5 eV. *Rev. Mod. Phys.* **40,** 737 (1968).
46. Guinier, A. *X-ray diffraction in crystals, imperfect crystals, and amorphous bodies*. (Dover Publications, 1994).
47. Ruan, C.-Y. *et al.* The development and applications of ultrafast electron nanocrystallography. *Microsc. Microanal.* **15,** 323–337 (2009).
48. Farrow, C. L., Ruan, C. Y. & Billinge, S. J. L. Quantitative nanoparticle structures from electron crystallography data. *Phys. Rev. B* **81,** 134104 (2010).
49. He, Z. & Millis, A. J. Photoinduced phase transitions in narrow-gap Mott insulators: The case of $VO_2$. *Phys. Rev. B* **93,** 115126 (2016).

50. Yuan, X., Zhang, W. & Zhang, P. Hole-lattice coupling and photoinduced insulator-metal transition in $VO_2$. *Phys. Rev. B* **88,** 35119 (2013).
51. Haverkort, M. W. *et al.* Orbital-assisted metal-insulator transition in $VO_2$. *Phys. Rev. Lett.* **95,** 196404 (2005).
52. Maurer, D., Leue, A., Heichele, R. & Müller, V. Elastic behavior near the metal-insulator transition of $VO_2$. *Phys. Rev. B* **60,** 13249–13252 (1999).
53. Stabile, A. a. *et al.* Separating electric field and thermal effects across the metal-insulator transition in vanadium oxide nanobeams. *Appl. Phys. Lett.* **107,** 13503 (2015).
54. Wu, B. *et al.* Electric-field-driven phase transition in vanadium dioxide. *Phys. Rev. B* **84,** 241410(R) (2011).
55. Zhou, Y. *et al.* Voltage-triggered ultrafast phase transition in vanadium dioxide switches. *IEEE Electron Device Lett.* **34,** 220–222 (2013).
56. Zhou, Y. & Ramanathan, S. Correlated electron materials and field effect transistors for logic : A review. *Crit. Rev. Solid State Mater. Sci.* **38,** (2013).
57. Rúa, A., Fernández, F. E. & Sepúlveda, N. Bending in $VO_2$ -coated microcantilevers suitable for thermally activated actuators. *J. Appl. Phys.* **107,** 74506 (2010).
58. Cabrera, R., Merced, E. & Sepúlveda, N. A micro-electro-mechanical memory based on the structural phase transition of $VO_2$. *Phys. Status Solidi A* **9,** 1704–1711 (2013).
59. Cao, J. *et al.* Strain engineering and one-dimensional organization of metal-insulator domains in single-crystal vanadium dioxide beams. *Nat. Nanotechnol.* **4,** 732–737 (2009).
60. Heavens, O. S. Optical properties of thin films. *Reports Prog. Phys.* **23,** 1–65 (1960).
61. Verleur, H. W., Barker, A. S. & Berglund, C. N. Optical properties of $VO_2$ between 0.25 and 5 eV. *Phys. Rev.* **172,** 788–798 (1968).
62. Ruan, C.-Y. *et al.* The development and applications of ultrafast electron nanocrystallography. *Microsc. Microanal.* **15,** 323–337 (2009).
63. Westman, S. Note on a phase transition in $VO_2$. *Acta Chem. Scand.* **15,** 217 (1961).
64. Andersson, G. Studies on Vanadium Oxides. II. The Crystal Structure of Vanadium Dioxide. *Acta Chem. Scand.* **10,** 623–628 (1956).
65. Brown, P. J., Fox, a. G., Maslen, E. N., O'Keefe, M. a. & Willis, B. T. M. in *International Tables for Crystallography* **C,** 554–595 (2006).
66. Jones, A. C., Berweger, S., Wei, J., Cobden, D. & Raschke, M. B. Nano-optical investigations of the metal-insulator phase behavior of individual $VO_2$ microcrystals. *Nano Lett.* **10,** 1574–1581 (2010).
